# Supplementary material for: Continuously-tunable light–matter coupling in optical microcavities with 2D semiconductors
Source: Sci Rep. 2020 May 19;10:8303. doi: 10.1038/s41598-020-64909-1 (PMC7237431; doi:10.1038/s41598-020-64909-1)
Supplement: Supplementary file 1 — Supplementary information. [file 41598_2020_64909_MOESM1_ESM.pdf]

Supporting Information (SI)

Continuously-tunable light–matter coupling in optical microcavities with 2D semiconductors

Franziska Wall, Oliver Mey, Lorenz Maximilian Schneider and Arash Rahimi-Iman*

Faculty of Physics and Materials Sciences Center, Philipps-Universität Marburg, D-35032 Marburg, Germany
**email: a.r-i@physik.uni-marburg.de*

Complex refractive index

In order to obtain the coupled-system's reflectivity spectra, the role of the transition-metal dichalcogenide monolayer inside the cavity is represented in our simulations by the complex refractive index extracted from the literature. Therefore, experimental data from Ref [42] is modelled in a reasonable approximation by a summation over multiple Lorentzian peaks according to Ref. [43].

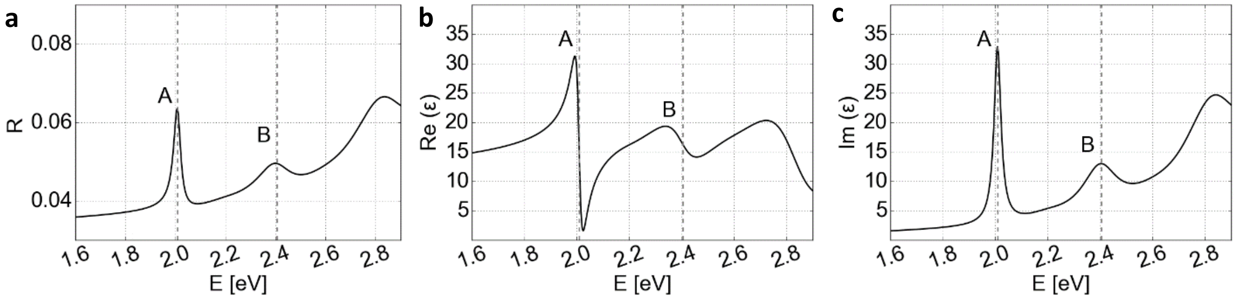

Fig. SI. 1: Simulated reflectivity of a WS_2 monolayer on top of an SiO_2 layer obtained by an approximation of experimental data from Ref. [40] with a summation over Lorentzian peaks according to Ref. [41] (a). Corresponding real and imaginary parts of the dielectric function, which are used here within the transfer-matrix method, are shown in (b) and (c), respectively. The vertical dashed lines mark the A and B exciton position.

Figure SI.1 shows the corresponding curve of the reflectivity (a) of a monolayer on top of silica with an optical thickness of 6.18 Å as well as the real (b) and imaginary part (c) of its dielectric function based on the data from [42] and [43]. Here, the exciton wavelength is determined by the peak position in the imaginary part of the dielectric function. The dependency of the dielectric function on the angle of incidence as well as on the light's polarisation is neglected in our simulation. For further investigations a detailed measurement of the angle-dependent reflectivity of a monolayer would be desired to get access to this information complemented by an angle-resolving theoretical modelling of the complex dielectric function. The in-plane permittivity $\epsilon_{||}$ of a WS_2 monolayer is larger than its out-of-plane $\epsilon_{\perp}$ [S1]. Thus, the resonant cavity length in out-of-plane direction is for $\vartheta = 90°$ roughly 1.44 times larger compared to the value estimated by the in-plane permittivity.

$$2\,\pi = \Delta s_{cav} = \sqrt{\epsilon}\, L_{cav} \cos{(\vartheta)}, \qquad \text{(SI 1)}$$

$$L_{cav,\perp} = \sqrt{\frac{\epsilon_{||}}{\epsilon_{\perp}}} L_{cav,||} \approx 1.44\, L_{cav,||}\,. \qquad \text{(SI 2)}$$

Further, for s-polarized light the out-of-plane exciton cannot couple with the light field, because its dipole moment is perpendicular to the electric field. Moreover, the out-of-plane exciton has an about 10^3 smaller oscillator strength compared to the in-plane exciton [S2]. Consequently, the coupling for out-of-plane components with p-polarized light is reduced by about 1.5 orders of magnitude compared to in-plane components. Thus, the effect of anisotropy is neglected in our considerations.

Transfer-matrix method

Optical properties of thin-film stacks can be modelled one-dimensionally by the transfer-matrix method. Here, we give a short overview of the applied formulas in our simulation code. A detailed description could be found e.g. in [S3-S5]. Plane waves travelling in the z direction perpendicular to the layer interfaces are described as superposition of one right propagating $\mathrm{E}_n^- = 0$ and one left propagating E_i^- wave in layer i, whereas each layer is assumed to be homogeneous.

$$E(z) = \mathrm{E}_i^+ \exp(ik_{i,z}z) + \mathrm{E}_i^- \exp(-ik_{i,z}z). \quad \text{(SI 3)}$$

Here, we chose the minus-sign inside of the exponential function for waves propagating to the structure's left side.

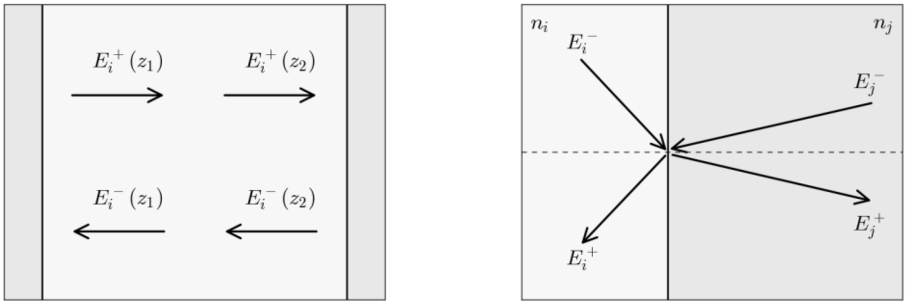

Fig.SI. 2: Schematic representation of the waves' propagation as well as behaviour at a layer interface which is used in the transfer matrix method.

The wave propagation inside of a layer i is described by a phase shift $\varphi_i = 2\pi d_i \cos(\vartheta_i)/\lambda$, which is determined by the travelled distance d_i and the wave vector $k_{i,z}$ in z direction. The later one is given by the wavelength λ, the angle of incidence ϑ_i and the material's complex refractive index n_i.

$$E_i^+(z = d_i) = exp(i\varphi_i)E_i^+(z = 0), \quad \text{(SI 4)}$$

$$E_i^-(z = 0) = exp(i\varphi_i)E_i^-(z = d_i). \quad \text{(SI 5)}$$

Using a matrix formalism, the phase matrix P_i is defined as following:

$$\begin{pmatrix} E_i^+(z=0) \\ E_i^-(z=0) \end{pmatrix} = \begin{pmatrix} exp(-i\varphi_i) & 0 \\ 0 & exp(i\varphi_i) \end{pmatrix} \begin{pmatrix} E_i^+(z=d_i) \\ E_i^-(z=d_i) \end{pmatrix} = P_i \begin{pmatrix} E_i^+(z=d_i) \\ E_i^-(z=d_i) \end{pmatrix}. \quad \text{(SI 6)}$$

A wave transmitted through the interface from layer i to layer j can be modelled by a reflection coefficient r_{ij} and transmission coefficient t_{ij}. The index' order represents thereby from which layer to which the wave is propagating.

$$E_j^+(z) = t_{ij}E_i^+(z) + r_{ji}E_j^-(z), \quad \text{(SI 7)}$$

$$E_j^-(z) = r_{ij}E_i^+(z) + t_{ji}E_j^-(z). \quad \text{(SI 8)}$$

The reflection and transmission coefficient are calculated by the Fresnel equations. In our case, the equation for s-polarized light is applied.

$$r_{ij,s} = \frac{E_i^-}{E_i^+} = \frac{n_i cos(\vartheta_i) - n_j cos(\vartheta_j)}{n_i cos(\vartheta_i) + n_j cos(\vartheta_j)} \;, \quad \text{(SI 9)}$$

$$t_{ij,s} = \frac{E_j^+}{E_i^+} = \frac{2 n_i cos(\vartheta_i)}{n_i cos(\vartheta_i) + n_j cos(\vartheta_j)} \;. \quad \text{(SI 10)}$$

Again, this can be summarized in the matrix form with the interface matrix T_{ij}. The later form of T_{ij} follows directly from the Fresnel equations by $r_{ij} = -r_{ji}$.

$$\begin{pmatrix} E_i^+(z) \\ E_i^-(z) \end{pmatrix} = \frac{1}{t_{ij}} \begin{pmatrix} 1 & r_{ij} \\ r_{ij} & 1 \end{pmatrix} \begin{pmatrix} E_j^+(z) \\ E_j^-(z) \end{pmatrix} = T_{ij} \begin{pmatrix} E_j^+(z) \\ E_j^-(z) \end{pmatrix}. \quad \text{(SI 11)}$$

Now, the complete structure can be modelled layer-by-layer by multiplication of the corresponding phase and interface matrices,

$$T_{structure} = T_{01}P_1 \ldots P_{n-1}T_{(n-1)n}. \quad \text{(SI 12)}$$

If the light incidents only from the left side of the structure ($E_n^- = 0$), the reflection and transmission coefficient of the complete structure follows the matrix elements s_{ij} of $T_{structure}$,

$$r_{0n} = \frac{E_0^-}{E_0^+} = \frac{s_{21}}{s_{11}} \;, \quad \text{(SI 13)}$$

$$t_{0n} = \frac{E_n^+}{E_0^+} = \frac{1}{s_{11}} \;. \quad \text{(SI 14)}$$

With this the reflection and transmission of the incident light is obtained.

$$R_{0n} = |r_{0n}|^2, \quad \text{(SI 15)}$$

$$T_{0n,s} = \frac{\Re(n_n cos(\vartheta_n))}{\Re(n_0 cos(\vartheta_0))} |t_{0n}|^2. \quad \text{(SI 16)}$$

Waves of different wavelength are simulated to obtain a spectrum. The waves' angle of incidence is varied for angle-resolved spectra.

To derive the field at position z, the right as well as left propagating wave at z are determined. The field at a layer interface serves as a starting point. Positions inside of one layer follow directly by using the phase matrix. Transfer coefficients τ_{ij} are defined as connection between the incident field E_0^+ with the inner field amplitude at a layer's interface (E_i^+ and E_i^-).

$$\frac{E_i(z)}{E_0^+} = E_i^+(z) + E_i^-(z) = \frac{E_i^+}{E_0^+} exp(-\varphi_i(z)) + \frac{E_i^-}{E_0^+} exp(\varphi_i(z)) = \tau_{0i,+} exp(-\varphi_i(z)) + \tau_{0i,-} exp(\varphi_i(z)) \quad . \quad \text{(SI 17)}$$

These coefficients can be expressed in terms of the transfer matrix $T^I = T_{0i}$ and $T^{II} = T_{in}$ with their matrix elements s_{ij}^I and s_{ij}^{II}, respectively.

$$\tau_{0i,+} = \frac{E_i^+}{E_0^+} = \frac{1}{det(T^I)}(s_{22}^I - r_{0n}s_{12}^I), \quad \text{(SI 18)}$$

$$\tau_{0i,-} = \frac{E_i^-}{E_0^+} = \frac{1}{det(T^I)}(r_{0n}s_{11}^I - s_{21}^I). \quad \text{(SI 19)}$$

The reflection coefficient r_{0n} can be expressed as well by s_{ij}^I and s_{ij}^{II}.

$$r_{0n} = \frac{E_0^-}{E_0^+} = \frac{s_{21}}{s_{11}} = \frac{s_{21}^I s_{11}^{II} exp(i\varphi_i(z)) + s_{22}^I s_{21}^{II} exp(-i\varphi_i(z))}{s_{11}^I s_{11}^{II} exp(i\varphi_i(z)) + s_{12}^I s_{21}^{II} exp(-i\varphi_i(z))}. \quad \text{(SI 20)}$$

Based on this, the following equation can be found to model the field at position z,

$$E_i(z) = \frac{s_{11}^{II} exp(-i\varphi(d_i - z)) + s_{21}^{II} exp(i\varphi(d_i - z))}{s_{11}^{II} s_{11}^I exp(i\varphi(d_i)) + s_{12}^I s_{21}^{II} exp(-i\varphi(d_i))} E_0^+. \quad \text{(SI 21)}$$

Phase-shift in a planar open microcavity with a spacing layer

Here, we further explain the phase shift inside of the cavity in order to give an expression to estimate the necessary angular change for a given cavity length change. The phase shift of the complete cavity consists of the phase shift at each mirror surface φ_{mirror}, the phase shift inside of the solid spacer layer (here PMMA) and the air layer, φ_{PMMA} and φ_{Air}, respectively.

$$\varphi_{cav} = \varphi_{mirror,1} + \varphi_{mirror,2} + \varphi_{PMMA} + \varphi_{Air}. \quad \text{(SI 22)}$$

In order to keep the cavity resonant to the exciton, this phase shift must remain constant for each mirror separation. Therefore, the change of the phase shift $\Delta\varphi_{cav}$ should be a multiple of 2π. For simplicity, the phase shift change at the mirror surface for a tuned angle of incidence is neglected in the following.

$$m\,2\pi = \Delta\varphi_{cav} = (\varphi_{PMMA}(L_{cav,1}) + \varphi_{Air}(L_{cav,1})) - (\varphi_{PMMA}(L_{cav,2}) + \varphi_{Air}(L_{cav,2})) =$$

$$= (n_{PMMA}d_{PMMA}cos(\vartheta_1) + n_{Air}d_{Air}cos(\vartheta_1)) - (n_{PMMA}d_{PMMA}cos(\vartheta_2) + n_{Air}(d_{Air} + \Delta z)cos(\vartheta_2)),$$

$$with\ m\epsilon\mathbb{N}\,. \quad \text{(SI 23)}$$

The connection between the current angle of incidence ϑ_2 and the change of the cavity length Δz is thus found by including the expression for the phase shift, $\varphi_i = 2\pi n_i d_i \cos(\vartheta_i)/\lambda$,

$$cos(\vartheta_2) = \frac{n_{PMMA}d_{PMMA} + n_{Air}d_{Air,1}}{n_{PMMA}d_{PMMA} + n_{Air}(d_{Air,1} + \Delta z)} cos(\vartheta_1). \quad \text{(SI 24)}$$

Thus, the connection between the change of the cavity length and the resonant angle can be mainly addressed through the choice of the thickness of the spacing layer and the air gap. The resonant angle differs for higher modes from that of the cavity's ground state (Fig. SI 3).

Connection between air gap and the resonant angle for different modes

The connection between the resonant angle of incidence θ_{res} *(*E_{cav} *(*θ_{res}*) -* E_{ex} *= 0)* and the open-cavity spacing Δz according to Equation (SI 24) is shown in Fig. SI.3 (a) for a 178.2 nm thick PMMA spacing layer. For comparison, the equivalent simulated relationship based on the transfer-matrix method is shown in Fig. SI.3b. For clarity it is noted that when PMMA is involved, the microcavity structure type corresponds to that of Fig. 1b. In Fig. SI.3 both graphs display the trend for the first resonant mode (blue solid line/triangles) of the cavity and the next higher mode (green dashed line/circles). The open-cavity spacing for the two consecutive resonator modes at normal incidence is thereby 48 nm and 356.5 nm, respectively. The phase shift change due to the angle-dependent reflectivity of the mirrors (and thereby

the angle dependency of φ_{mirror}) is neglected in Equation (SI 24) and leads to the differences between the two plots.

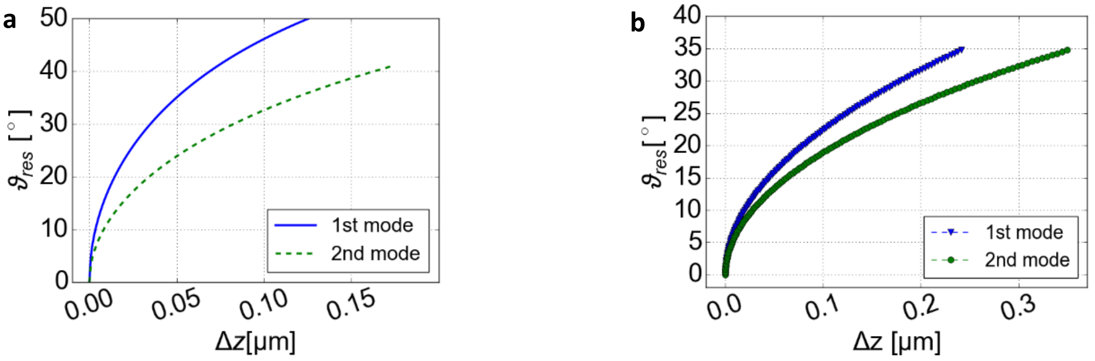

Fig. SI. 3: (a) Resonant angle of incidence as a function of the spacing distance Δz for the fundamental and second resonator mode according to Eq. (SI 22). (b) Equivalent trends based on the transfer-matrix-method simulation for a representative configuration.

Relative field strength for higher resonant modes

To show the influence of the mode number on the standing-wave pattern at resonance conditions for three consecutive modes, i.e. the fundamental resonator modes and the two higher ones, the calculated relative field strength as well as the angle-resolved reflectivity spectra are plotted in Fig. SI.4. The simulations were performed for the case of an 178.2 nm thick PMMA layer on top of the left DBR and a resonant angle of incidence ϑ_{res} of 5° corresponding to air gaps for the three different modes of 51.0 nm, 360.8 nm and 670.5 nm, respectively. Here, similar to the considerations regarding the graphs in Fig. 4, only the reflectivity of the 2D-material is considered but not its absorption (the result in (b) corresponds to that in Fig. 4a shown for the second resonator mode). The splitting within the reflectivity spectra is smaller for higher modes due to the smaller mode volume.

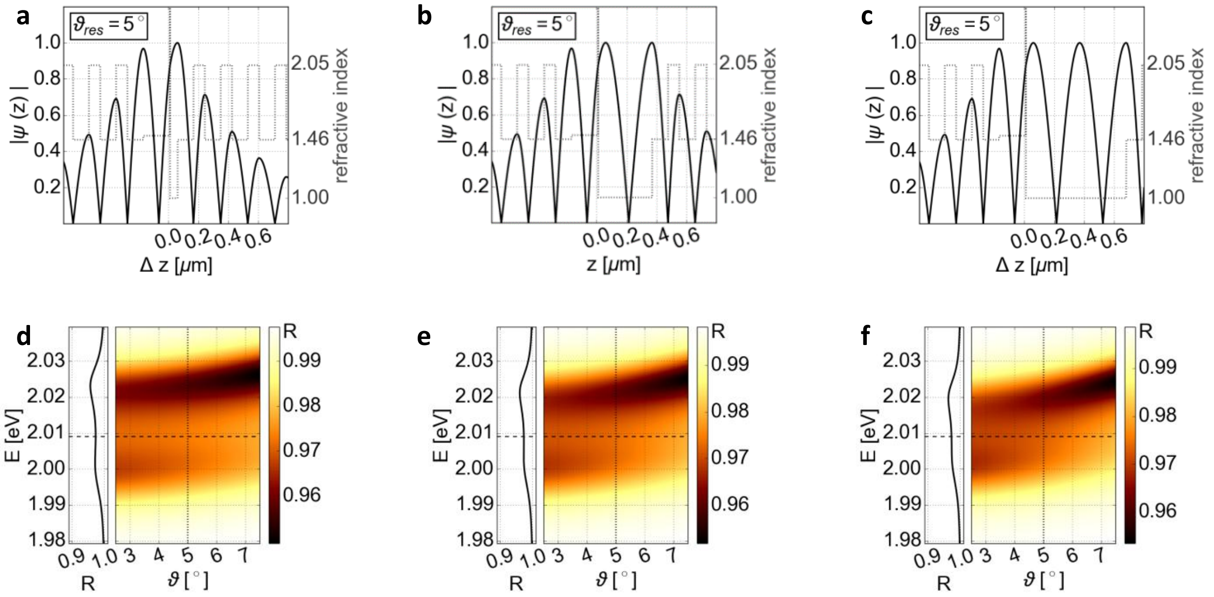

Fig. SI. 4: Theoretical relative field strength for the first cavity mode (a), the second (b) and the third resonator mode (c) for an open microcavity structure with an angle of incidence of 5° corresponding to that in Fig. 1b. The PMMA layer on top of the left DBR is 178.2 nm thick and the air gaps are 51.0 nm,

360.8 nm and 670.5 nm for the first three modes, respectively. (d-f) show the corresponding calculated angle-dependent reflectivity for the different cases similar to Fig. 4.

Comparison of the coupling situation for two resonant modes

For the ground state and the energetically next higher cavity mode, the relationship between the cavity's length change and the necessary resonant angle of incidence ϑ_{res} differs because the air gap of the higher mode is larger (Equation SI 1, Fig. SI. 2). Thus, for a higher mode, a larger change of the mirror separation is necessary to reach the same shift for the resonant angle. On the other hand, the same angular change leads to the same modification of the relative field strength at the exciton position. Altogether, the splitting in our simulation is higher for the first resonant mode due to the smaller mode volume (Fig. SI.5).

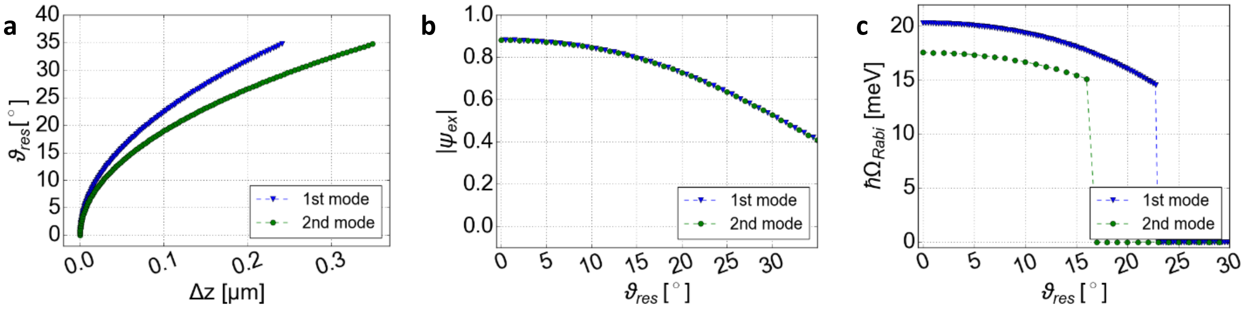

Fig. SI.5: Relationship between the cavity length change Δz and the resonant angle ϑ_{res} (a), the relative field strength (b) and the resolvable splitting $\hbar\Omega_{Rabi}$ within the reflectivity spectrum (c) for the fundamental resonant cavity mode as well as the energetically next higher mode for a structure with a 178.2 nm thick PMMA spacing layer. In (b) and (c), ϑ_{res} is directly linked with the corresponding Δz in (a).

Determination of the resonant angle

The resonant angle at a given cavity length is determined iteratively until spectral overlap between the cavity mode and the exciton's emission wavelength within the calculated reflectivity spectrum is reached, while the absorption of WS_2 is neglected (representing an uncoupled situation). For a 178.2 nm thick PMMA spacer layer and an air gap of 356.5 nm, this is the case for normal incidence shown in Fig. SI.6a. The split between the normal modes in the calculated reflectivity with included absorption of WS_2, which represents the spectrum in the coupled situation, is slightly asymmetric, as can be seen in Fig. SI.6b, although the "empty" (uncoupled) cavity mode is energetically resonant with the exciton energy.

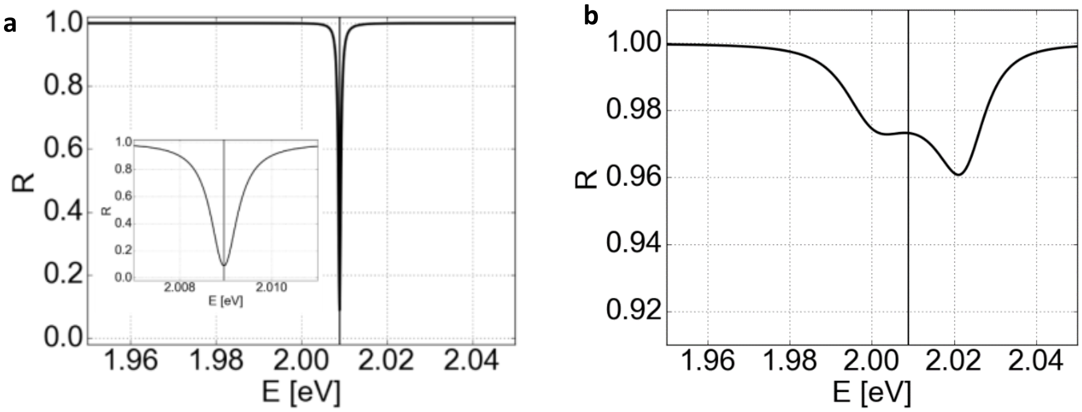

Fig. SI. 6: Calculated reflectivity spectrum for an uncoupled cavity with inserted WS_2 (a). Inset: close-up spectrum of the resonance. In comparison, the coupled cavity shows a mode splitting in the calculated reflection spectrum with included absorption of WS_2 (b). The slight asymmetry at determined resonance condition is attributed to the role of the absorbing layer on the actual cavity field configuration, which is approximated for the microcavity system when neglecting the imaginary part of the dielectric function. The extracted resonance energy from (a) is indicated by the vertical line in both graphs.

Angle resolved reflectivity of the DBR mirror

The angle-resolved reflectivity of a SiO_2/Si_3N_4 DBR with 11.5 layer pairs according to transfer-matrix-method calculations is depicted in Fig. SI.7a. Over the explored range of angles (smaller than 40° in this study) the reflectivity change as a function of incidence angle is less than 0.1%, which will have a minor impact on the cavity Q-factor for different angles. Fig. SI.7 shows that the field distribution at normal incidence (b) has a maximum at the DBR's surface, whereas for an angle of 35° this maximum is shifted (c). Thus, the DBR contributes an additional phase shift at higher angles of incidence.

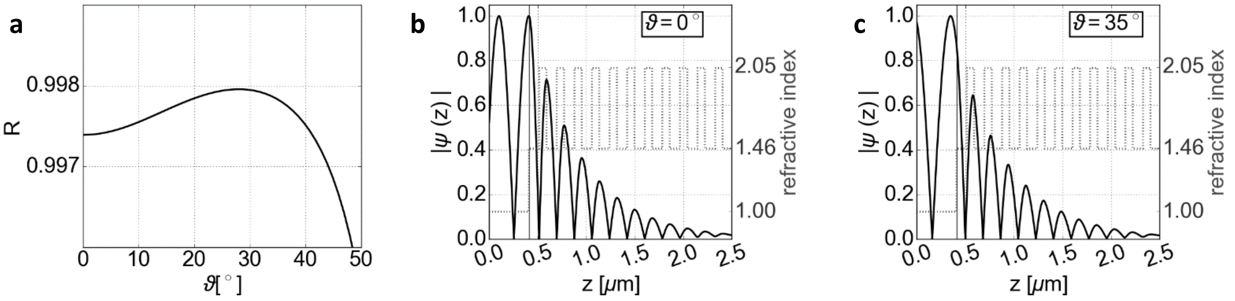

Fig. SI.7: (a) Calculated DBR reflectivity as a function of the incidence angle. For comparison, the field distribution in the vicinity of the DBR surface is shown for normal incidence (b) and a 35 degree incidence angle (c).

Angle resolved transmission and absorption spectra for s-polarized light

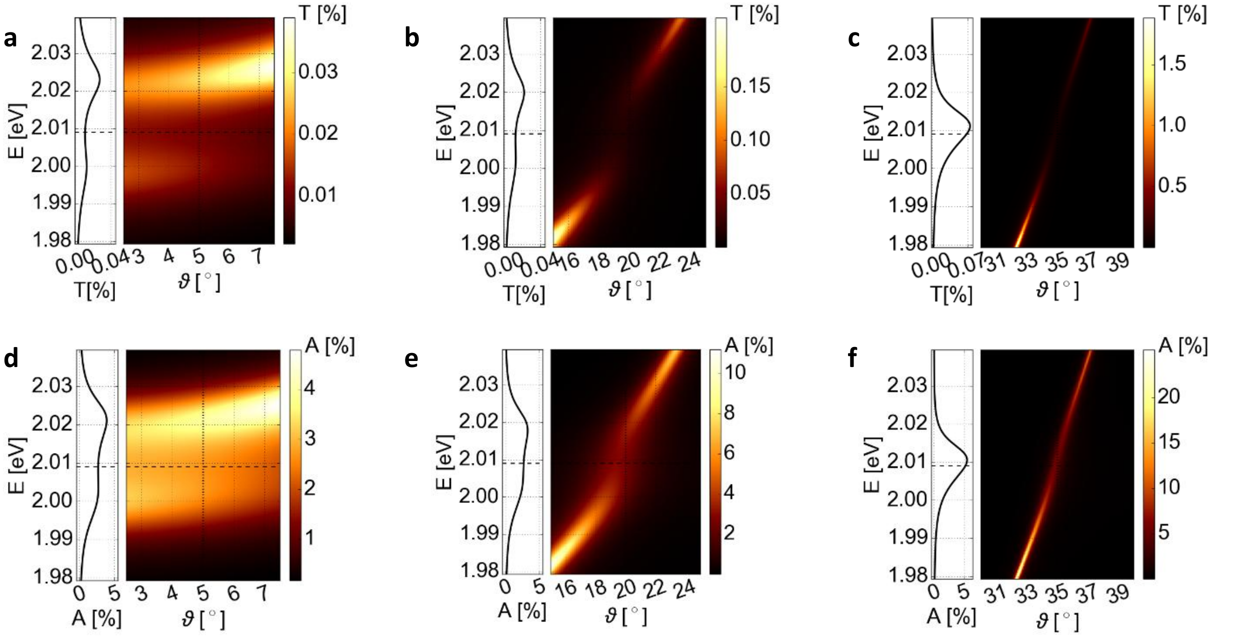

Fig. SI.8: Influence on the transmission (upper row) and absorption (lower row) for s-polarized light by a tuned cavity length. The dashed vertical line marks the exciton's emission energy, which is identified by the peak position within the imaginary part of the refractive index. Within the absorption spectra, the splitting is nearly symmetric. As expected from literature, the splitting in reflection, transmission and absorption differs [S6]. Note, that the observed splittings depend on the cavity's as well as exciton's linewidth.

For the reflectivity calculations provided in Fig. 4, corresponding angle-resolved transmission and absorption spectra are summarized in Fig. SI.8. For the microcavity structure type of Fig. 1b, the spacer layer amounts to 178.2 nm in the calculated examples. For the structure's air gap of 360.8 nm, 425.6 nm and 575.7 nm, the resonant angle lies at 5°, 20° and 35°, respectively.

Angle resolved reflectivity, transmission and absorption spectra for p-polarized light

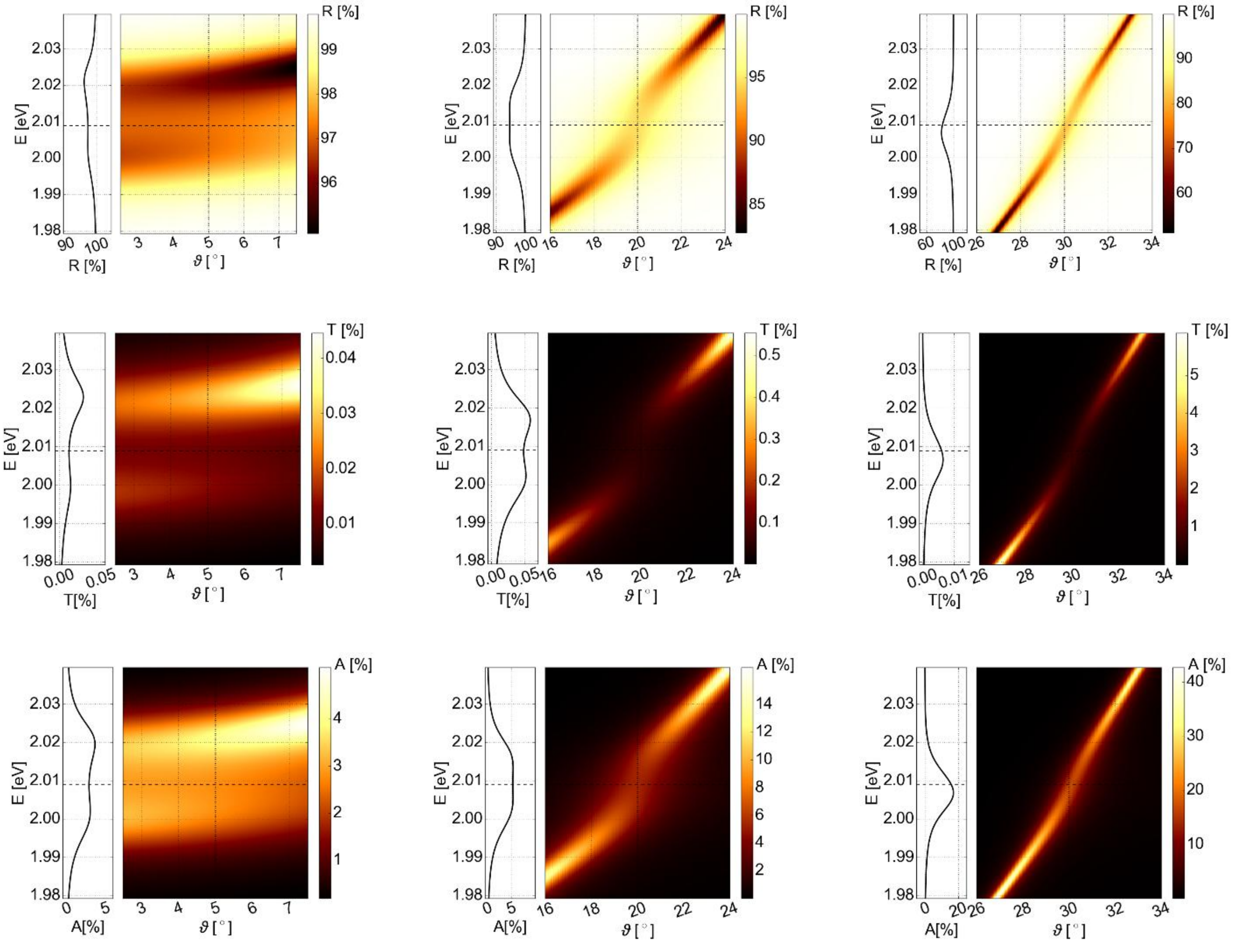

Fig. SI.9: Impact on the reflection (upper row), transmission (middle row) and absorption (lower row) for p-polarized light by a tuned cavity length. The dashed vertical line marks the exciton's emission energy, which is identified by the peak position within the imaginary part of the refractive index.

Figure SI.9 shows the influence on the transmission (upper row) and absorption (lower row) for p-polarised light of a tuned cavity length with a 178.2 nm thick PMMA spacer layer on one mirror (structure depicted in Fig.1b). The structure's air gap is 360.8 nm, 425.6 nm and 515.0 nm with a resonant angle at 5.08°, 20.315° and 35.008°, respectively. The air gap corresponds thereby to resonant angles for s-

polarized light of 5°, 20° and 35°, respectively. Thus, the determined resonant angles for s and p polarized light have only a small deviation from each other. The exciton's emission energy is represented by the dashed vertical line. The spectral detuning of the cavity mode by the exciton's absorption differs compared to s-polarized light. The overall effect of a continuously tunable coupling is nevertheless also observable for p-polarized light. Unpolarized light will lead to a weighted sum of the p- and s-polarized spectra.

Supporting Information References

[S1] Akash L., Van de Put M. L. & Vandenberghe W. G., Dielectric properties of hexagonal boron nitride and transition metal dichalcogenides: from monolayer to bulk. *npj 2D Materials and Applications* 2, 6, (2018).

[S2] Wang, G., Robert C., Glazov M., Cadiz F., Courtade E., Amand T., Lagarde D., Taniguchi T., Watanbe K., Urbaszek, B. & Marie X., In-Plane Propagation of Light in Transition Metal Dichalcogenide Monolayers: Optical Selection Rules. *Phys. Rev. Lett.* 119, 047401, (2017)

[S3] Z. KNITTL, Optics of thin films: (an optical multilayer theory), Wiley series in pure and applied optics, Wiley, London, 1976.

[S4] Pettersson, L. A. A., Roman, L. S. & Inganäs, O. Modeling photocurrent action spectra of photovoltaic devices based on organic thin films. Journal of Applied Physics 86, 487–496; 10.1063/1.370757 (1999).

[S5] Kim, J., Jung, S. & Jeong, I. Optical Modeling for Polarization-dependent Optical Power Dissipation of Thin-film Organic Solar Cells at Oblique Incidence. Journal of the Optical Society of Korea 16, 6–12; 10.3807/JOSK.2012.16.1.006 (2012).

[S6] Savona, V., Andreani, L.C., Schwendimann, P. & Quattropani, A. Quantum well excitons in semiconductor microcavities. Unified treatment of weak and strong coupling regimes. *Solid State Communications* **93**, 733-739; 10.1016/0038-1098(94)00865-5 (1995).
