# Supplementary figures and images for: Continuously-tunable light–matter coupling in optical microcavities with 2D semiconductors
Source: Sci Rep. 2020 May 19;10:8303. doi: 10.1038/s41598-020-64909-1 (PMC7237431; doi:10.1038/s41598-020-64909-1)

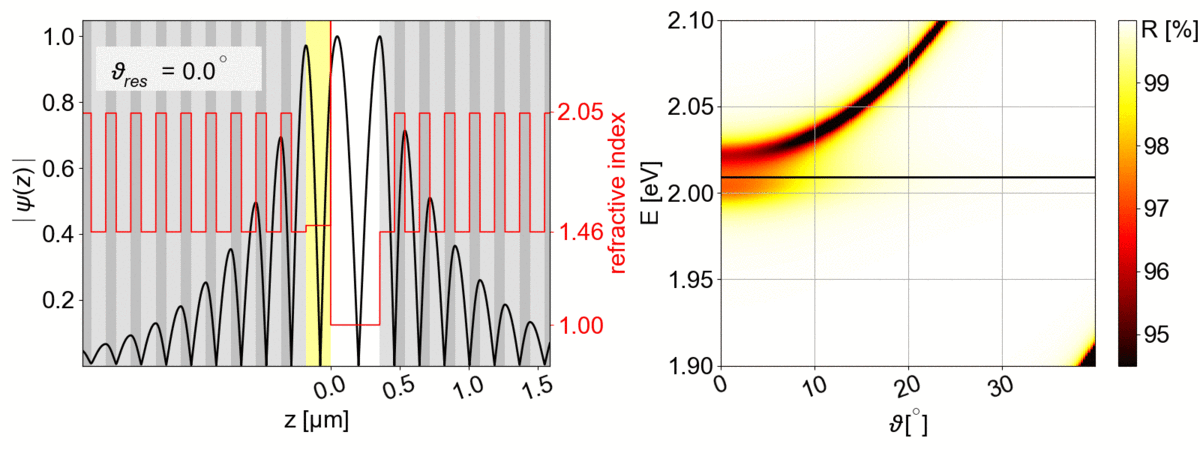

Supplement: Supplementary file 2 — Supplementary Video [file 41598_2020_64909_MOESM2_ESM.gif]
